# Isolation, characterization, and application of a novel polyvalent lytic phage STWB21 against typhoidal and nontyphoidal *Salmonella* spp.

**DOI:** 10.3389/fmicb.2022.980025

**Published:** 2022-08-22

**Authors:** Payel Mondal, Bani Mallick, Moumita Dutta, Shanta Dutta

**Affiliations:** ^1^Division of Electron Microscopy, ICMR-National Institute of Cholera and Enteric Diseases, Kolkata, West Bengal, India; ^2^Division of Bacteriology, ICMR-National Institute of Cholera and Enteric Diseases, Kolkata, West Bengal, India

**Keywords:** *Salmonella*, lytic, bacteriophage, biofilm, onion

## Abstract

*Salmonella* is one of the common causal agents of bacterial gastroenteritis-related morbidity and mortality among children below 5 years and the elderly populations. Salmonellosis in humans is caused mainly by consuming contaminated food originating from animals. The genus *Salmonella* has several serovars, and many of them are recently reported to be resistant to multiple drugs. Therefore, isolation of lytic *Salmonella* bacteriophages in search of bactericidal activity has received importance. In this study, a *Salmonella* phage STWB21 was isolated from a lake water sample and found to be a novel lytic phage with promising potential against the host bacteria *Salmonella typhi.* However, some polyvalence was observed in their broad host range. In addition to *S. typhi,* the phage STWB21 was able to infect *S. paratyphi, S. typhimurium, S. enteritidis,* and a few other bacterial species such as *Sh. flexneri 2a, Sh. flexneri 3a,* and *ETEC.* The newly isolated phage STWB21 belongs to the *Siphoviridae* family with an icosahedral head and a long flexible non-contractile tail. Phage STWB21 is relatively stable under a wide range of pH (4–11) and temperatures (4°C–50°C) for different *Salmonella* serovars. The latent period and burst size of phage STWB21 against *S. typhi* were 25 min and 161 plaque-forming units per cell. Since *Salmonella* is a foodborne pathogen, the phage STWB21 was applied to treat a 24 h biofilm formed in onion and milk under laboratory conditions. A significant reduction was observed in the bacterial population of *S. typhi* biofilm in both cases. Phage STWB21 contained a dsDNA of 112,834 bp in length, and the GC content was 40.37%. Also, genomic analysis confirmed the presence of lytic genes and the absence of any lysogeny or toxin genes. Overall, the present study reveals phage STWB21 has a promising ability to be used as a biocontrol agent of *Salmonella* spp. and proposes its application in food industries.

## Introduction

*Salmonella,* a member of the family *Enterobacteriaceae,* is the causative agent for gastro enteropathy and enteric (typhoid) fever (Reeves et al., 1989). Antibiotic resistant *Salmonella* spp. responsible for community-acquired infections are in the WHO high priority list of pathogens (Tacconelli et al., 2018). According to a recent report, *Salmonella* causes 115 million cases of an acquired bacterial food-borne illness named Salmonellosis and 370 thousand deaths every year globally (Seif et al., 2018). *Salmonella enterica* is a diverse species of bacteria consisting of more than 2,600 different serovars and it is one of the serious pathogens to human health as well as in animals (Grimont and Weill, 2007; Gal-Mor et al., 2014). *Salmonella enterica* spp. is divided into two main groups, typhoidal *Salmonella* (TS) and non-typhoidal *Salmonella* (NTS). Typhoidal *Salmonella* being highly adapted to the human host causes life-threatening enteric fever whereas gastroenteritis, and bacteremia are caused by non-typhoidal *Salmonella* (Gal-Mor et al., 2014). Non-typhoidal *Salmonella* which is mostly zoonotic can manifest disease in two forms invasive and non-invasive (Hohmann, 2001). Also, different food sources like vegetables, fruits, milk, chicken, beef or the consumption of raw or undercooked foods, etc. were identified as vehicles of *Salmonella*-associated foodborne outbreaks in the past decade (Carrasco et al., 2012; Devleesschauwer et al., 2015).

Both in nature and disease state, some bacteria create a surface-associated community known as biofilm for long-term survival in hostile environments. Additionally, bacteria within a biofilm are more prone to resistance to antibiotics compared to those in planktonic bacterial cells (Hall and Mah, 2017). Therefore, the biofilm-mediated infection has become a public health concern. For treatment purposes, newly synthesized drugs and combinational therapy have been developed but satisfactory results are yet to be achieved. *Salmonella* is one of the commonly known bacterial species that form biofilms (Done et al., 2015). It has also been reported that bacterial biofilm can significantly contribute to pathogenesis by creating resistance against the body’s defense system (Bai et al., 2021). In fact, the capability of *Salmonella* spp. to form biofilm is generally connected with their pathogenicity (Vestby et al., 2020). Bacteriophages have some unique properties that allow the treatment of biofilms effectively. There are several reports on the anti-biofilm properties of lytic phages both *in vitro* and in clinical infection (Azeredo and Sutherland, 2008; Doub, 2020).

Bacteriophages are naturally-occurring bacterial viruses, that can specifically infect and lyse a bacterial cell without affecting the host microflora (Housby and Mann, 2009). The specific antibacterial activities of lytic phages hint at their applications as alternatives to antibiotic therapy for humans and animals (Thung et al., 2017). However, some polyvalence is predominately observed among phages of Enterobacteriaceae at the beginning of the last century (Parra and Robeson, 2016). Additionally, the polyvalent phages are able to infect a broad host spectrum of bacteria from either different genera or species (Hamdi et al., 2017). Furthermore, it has been hypothesized that phages isolated from one region might not infect bacteria in other regions due to the broad diversity and defense mechanisms of enteric bacteria. Therefore, a continuous search of new phages focussing on different serovars with high lytic ability is certainly required for practical application (Kropinski et al., 2009). In phage-bacterial interactions, physicochemical factors such as temperature and pH play an essential role. Therapeutic phages are generally selected based on large burst size, short latent period, high adsorption rate, broad host range, strong antimicrobial property, and lytic capacity for further applications (Nilsson, 2019).

In this study, we aimed to isolate and characterize a novel polyvalent bacteriophage STWB21 with infectivity against different typhoidal and non-typhoidal *Salmonella* serovars for its potential use in phage therapy. We investigated the microbiological and morphological properties, genomic and proteomic analysis, and lytic activity of phage against biofilms under controlled conditions *in vitro*.

## Materials and methods

### Bacterial strains

The *Salmonella* bacterial strains used in this study were isolated from the patient’s blood sample at the Division of Bacteriology, ICMR-NICED, India. The bacterial strains were grown under aerobic conditions in Luria-Bertani (LB) broth at 37°C following institutional standard biosafety guidelines.

### Isolation and purification of bacteriophage

The water sample was collected from lake water in the outskirt area about 18 km from Kolkata, West Bengal, India in search of phage isolation. Briefly, 25 ml of water sample was mixed with 25 ml of 10X phage broth media and 5 ml of log-phase *Salmonella typhi* culture and incubated at 37°C for 24 h under shaking conditions at 100 rpm. The residual bacterial cells were removed by centrifugation at 10,000 rpm for 10 min at 4°C and the supernatant was filtered with a 0.22 μm membrane filter. To confirm the presence of the phage in the filtrate, a spot assay was performed on *Salmonella typhi* using the double-layer agar method (Sambrook and Russell, 2001; Kutter, 2009). Then the single plaque was picked up three times by plaque assay to obtain the purified phage plaque. After proliferation, the phage was purified and concentrated by ultracentrifugation (25,000 rpm, 1.5 h, 4°C) and sucrose step-gradient ultracentrifugation (30,000 rpm, 2 h, 4°C) respectively (Dutta and Ghosh, 2007).

### Host range and efficiency of plating

Host range assay was performed to evaluate the lytic spectrum of the obtained bacteriophages to the susceptible bacterial strains. It was determined by spotting 10 μl of phage lysate (10^10^ PFU/ml) on each agar plate with different bacterial strains. The plates were incubated at 37°C overnight and examined for clear zones that indicate the susceptibility of the bacteria. Further, an EOP assay of the obtained phage was then conducted by the double-layer agar method to quantify the lytic activity of phage STWB21.

### Transmission electron microscopy

3 μl purified phage lysate (10^10^ pfu/ml) was applied to a carbon-coated copper grid and negatively stained with 2% (w/v) uranyl acetate for 30 s and excess liquid was blotted off (Czajkowski et al., 2015). After air-drying the grids were visualized with FEI Tecnai 12 BioTwin Transmission Electron Microscope operating at 100 kV.

### Stability

A thermal stability test of phage STWB21 was carried out at pH 7.0 in order to investigate the heat-resistant capability of the phage. The phage was incubated at different temperatures (4°C, 25°C, 37°C, 50°C and 70°C respectively) for 1 h. After the incubation, the phage titer was determined by the soft-agar overlay method (Yuan et al., 2015). The pH stability of phage STWB21 was examined by pre-incubating the phage suspensions at a range of pH (pH 3–13) at 37°C for 1 h. Then the surviving phages were immediately counted by the double-layer agar method. Each assay was performed in triplicate.

### Adsorption assay and one-step growth assay

The adsorption assay was conducted as described previously with some modifications (Lu et al., 2003). Briefly, the bacterial culture (1 ml) was infected with a phage suspension at an MOI of 0.1 and incubated for 20 min at 37°C. After infection, aliquots were taken at different time intervals (0, 5, 10, 15, and 20 min) and immediately diluted in Tris-MgCl_2_ buffer, followed by centrifugation at 8,000 rpm for 5 min. Then the supernatant un-adsorbed phage titers were estimated by the double-layer agar method. In addition, a one-step growth curve of bacteriophage STWB21 was performed to access the growth kinetics of phage STWB21. It was performed according to the method described previously (Bloch et al., 2013). At an interval of 10 min, the exponentially growing bacterial culture (20 ml) was harvested and the pellet was resuspended in around 1 ml Luria broth followed by the addition of phage. The mixture was incubated for adsorption at 37°C for 5 min. The mixture was collected and immediately plated by the double-layer agar method. Burst size was calculated as the ratio of the final count of liberated phage particles to the initial count of infected bacterial cells during the latent period.

### Biofilm formation assay and effect of phage against biofilm

#### On glass coverslip

The biofilm degradation activity of newly isolated phage STWB21 on *S. typhi* was accessed following a previously discussed method with some modifications (Cerca et al., 2006; Nickerson et al., 2017; Mallick et al., 2021). A 10 μl of an overnight culture of *S. typhi* was dispensed into a Petri dish containing glass coverslips (22 mm × 22 mm) and incubated at 37°C for 24 h to form the biofilm. Two sets of Petri dishes were incubated for biofilm formation. One set was treated with buffer after 24 h incubation and the other set was treated with phage STWB21 overnight at 37°C.

### Application of bacteriophage in food items for controlling *Salmonella typhi*

#### On onion

The fresh onion bulb was chosen due to its association with foodborne illness outbreaks and variability in its surface structure. On the day of the experiment, fresh onion was purchased. Before an experiment, the onion was washed thoroughly for 5 min under running tap water (room temperature) followed by 70% alcohol to remove any soil or organic matter and any microflora present. After that, the cleaned onion bulb was placed in the center of a sterile Petri dish. Using a sterile scalpel, outer membranes were removed and the remaining scales of the onion were cut into 3 × 3 cm pieces. Further, these onion pieces were inoculated with 200 μl of *S. typhi* at 10^8^ CFU/ml and incubated at 37°C. After overnight incubation, the onion was treated with 20 μl of purified phage solution (1.5 × 10^10^ PFU/ml) for 4 h.

#### In milk

To study the removal and degradation of biofilm in milk by phage STWB21, we used pasteurized milk as the culture medium for the *S. typhi* biofilm. We purchased commercial sterile ultra-heat-temperature (UHT) treated milk from local retail. A logarithmic-growth-phase culture of *S. typhi* at a final concentration of 10^6^ CFU/ml was added to 1 ml of milk in two different sets of Petri dish containing glass coverslips (22 mm × 22 mm) to form the biofilm when incubated at 37°C for 24 h. After overnight incubation, one set of Petri dishes was treated with 40 μl of purified phage solution (1.5 × 10^10^ PFU/ml) for 4 h.

### Characterization of biofilm using scanning electron microscopy analysis

Biofilm samples of control and phage-treated groups were prepared for SEM analysis as described by Jahid et al. with some modifications (Jahid et al., 2013). Briefly, samples were fixed with 2.5% glutaraldehyde (Sigma-Aldrich, United States) in 0.1 M sodium cacodylate buffer at 4°C overnight. After that, the samples were serially dehydrated with ethanol (30%, 50%, 70%, and 90% for 10 min each, and 100% two times for 15 min each) and then successively treated with 100% hexamethyldisilazane (Sigma-Aldrich, United States) for 1 h. The dehydrated samples were sputter-coated with gold and visualized on FEI Quanta 200 SEM (FEI, Netherlands).

### Microtiter plate-based assay of biofilm

To evaluate the anti-biofilm efficacy of phage STWB21, biofilm was developed in 96-well plates in accordance with the previously described method with some modifications (Cerca et al., 2006). Briefly, the *S. typhi* strain was grown overnight at 37°C and was diluted to 1:100 in a fresh LB medium. Then 200 μl of the diluted culture was added to 96-well plates and was placed in an incubator at 37°C for 24 h without agitation. After incubation, the supernatant was removed from the well plate. To examine the effect of phage STWB21 on biofilm, phage stock was added into the biofilm at different concentrations (MOI 0.1 and 0.01) and incubated at 37°C for 4 and 24 h, respectively. Thereafter, the wells were rinsed with 1X PBS thrice and allowed to air-dry. The air-dried plate was then stained with crystal violet (0.1%, w/v) for 30 min. The optical densities of the biofilm were measured on the microplate reader at the absorbance of 595 nm (iMark Microplate Reader S/N 21673).

### SDS page analysis

To analyze the phage STWB21 protein profile, phage solution was boiled for 5 min and the structural proteins were extracted. The denatured proteins of phage STWB21 were separated by using 12.5% SDS-polyacrylamide gel electrophoresis (SDS-PAGE) as described by Laemmli (1970) with Mini-PROTEAN TGX Precast Gels (Bio-Rad, United States). After electrophoresis, proteins were visualized by staining with Coomassie Brilliant Blue.

### Proteomic analysis

Protein extraction was prepared as described in the SDS-PAGE assay. A total of 100 μg of protein was extracted and lyophilized. The 20 μg lyophilized protein was re-dissolved in 1.5 ml ice-cold 1 mM HCl (13 ng/μl trypsin prepared) and 100 μl aliquots stored at −20°C for further protein analysis. Protein in-solution digestion was performed at C-CAMP (NCBS, India) according to the previously described method (Wiese et al., 2007). Proteomic analysis was performed on 1,200, 1D nano-LC (Agilent Technologies, San Diego) that was coupled to Nanomate Triversa (Advion) and LTQ – Orbitrap Discovery (Thermo Fisher Scientific, United States). Nano LC-ESI-MS/MS spectra were searched using a PEAKS engine against phage genomes. For protein identification, the following parameters were used. Peptide mass Error tolerance = 10.0 ppm, fragment mass error tolerance = 0.6 Da, enzyme = trypsin, missed cleavage = 2, fixed modification: carbamidomethyl (C), variable modifications: oxidation (M), deamidation (NQ).

### Genomic DNA extraction and restriction digestion

DNA was extracted from the high-titer stocks of *Salmonella* phage STWB21 using a phage DNA isolation kit (Norgen Biotek Corp., Canada) according to the manufacturer’s instructions. The purity and concentration of the DNA were determined using a spectrophotometer. Then the DNA was digested by seven restriction enzymes namely: EcoRI, EcoRV, MluI, BglII, PstI, BamHI, and HindIII. Restriction fragments were separated by electrophoresis on 1.0% agarose gel and stained with ethidium bromide. DNA molecular weight marker (high range DNA ladder, HiMedia; ranging from 250 bp to 25 kbp) was used for the size determination of DNA fragments.

### Genomic characterization of phage STWB21

Purified genomic DNA of phage STWB21 was then sequenced by using an Illumina Platform at Xcelris (Ahmedabad, India). Reads were trimmed with Bioedit (version 7.1) and assembled with CLC Genomics Workbench v.6.0.5 with the reads map back option. The putative ORFs were predicted and annotated by GeneMark.hmm version 3.25^1^ and further confirmed by the RAST (McNair et al., 2018) server and tRNAs carried by the phage genomes were detected using the protein ARAGORN (Laslett and Canback, 2004) and tRNAscan-SE (Lowe and Eddy, 1997).^2^ Visualization of genome alignments of phage STWB21 with closely related *Siphovirida*e *Salmonella* phages was performed using the Easyfig application.^3^

To construct the phylogenetic tree, amino acid sequences of the terminase large subunits (ORF 130) and major capsid protein (ORF137) were selected and obtained from the NCBI Genbank database.^4^ Additionally, the terminase large subunits are usually considered as genetic marker (Lee et al., 2021) for the order Caudovirales and the major capsid protein is a highly conserved protein (Oh et al., 2014). The two phylogenetic trees were constructed with the default pipeline “ONE CLICK” at Phylogeny.fr (Dereeper et al., 2008).

### Statistical analyses

The statistical analysis was performed by *t*-test using GraphPad Prism (version 5, GraphPad Software, United States) software. All the values were tabulated as mean ± SD, and a significant difference between variations denoted by value of p were estimated using two-way ANOVA.

## Results

### Isolation, purification, and host range determination

The phage STWB21 was isolated from a lake water sample in the outskirts of Kolkata area. The host spectrum of phage STWB21 exhibited specific lytic activity against the prevalent typhoidal and non-typhoidal *Salmonella* spp., *Sh. flexneri* 2a, *Sh. flexneri* 3*a and ETEC* (Table 1). Phage STWB21 was found to be a novel polyvalent phage with high lytic activity against both typhoidal and non-typhoidal *Salmonella*. However, the phage did not show infectivity against some other species: *Sh. flexneri6, Sh. boydii* and *V. Cholerae 01* were used in this study. During infecting the prevalent *Salmonella* serovars, phage STWB21 formed clear plaques against *S. typhi, S. paratyphi, S. typhimurium* and *S. enteritidis*. The higher EOP values were obtained for the phage-sensitive *Salmonella* strains compared to the *Shigella* spp. and *ETEC* strains (Supplementary Table S1).

**Table 1:** Host range analysis of phage STWB21.

Strains	No. of strains used	No. of strains susceptible
1. *S. typhi*	4	4
2. *S. paratyphi*	3	3
3. *S. enteritidis*	1	1
4. *S. typhimurium*	1	1
5. *ETEC*	3	2
6. *Sh. flexneri 2a*	1	1
7. *Sh. flexneri 3a*	1	1
8. *Sh. flexneri 6*	1	0
9. *Sh. boydii*	1	0
10. *V. cholerae O1*	1	0

### Virion morphology

**Figure 1:**
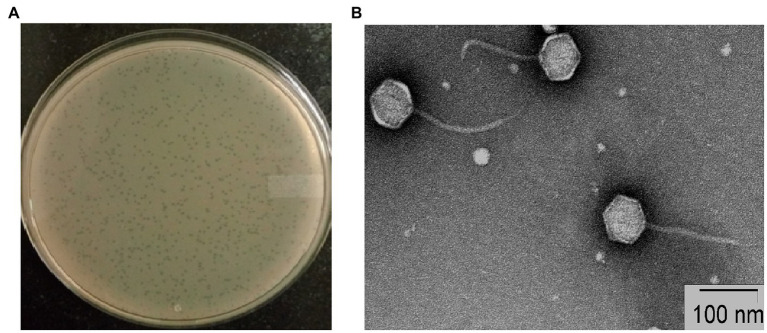
Morphology of bacteriophage STWB21. **(A)** Plaque morphology of phage STWB21, **(B)** transmission electron microscopic morphological study of phage STWB21.

Plaques of phage STWB21 on *S. typhi* bacterial lawn were measured around 1 mm diameter after overnight incubation (Figure 1A). Transmission Electron Microscopic study of phage STWB21 revealed a structure comprising an icosahedral head with an estimated diameter of 65 ± 3 nm (n = 20) and a long flexible, non-contractile tail of 113 ± 6 nm in length (*n* = 20) approximately (Figure 1B). Therefore, taking into account the morphological characteristics and criteria of ICTV (Fauquet and Fargette, 2005), phage STWB21 was classified within the *Caudovirales* order as a member of the *Siphoviridae* family (Ackermann, 2007).

### Phage stability test

**Figure 2:**
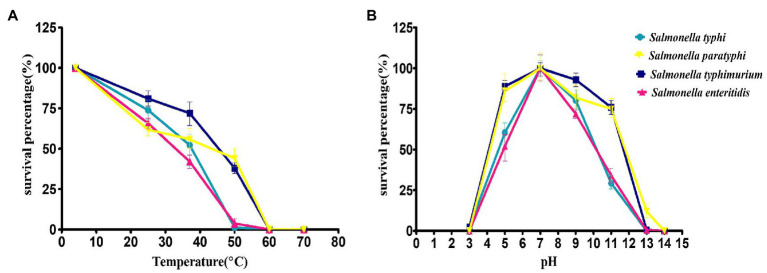
Biological characteristics of phage STWB21. **(A)** Thermal stability, **(B)** pH stability. Given values are the mean of three determinations.

The thermal stability of the phage STWB21 was analyzed on different typhoidal and non-typhoidal *Salmonella* spp. The phage STWB21 was remained stable up to 40°C for 1 h, but significantly decreased and lost its lytic activity at 50°C against the typhoidal and non-typhoidal host strains (Figure 2A). On the other hand, phage STWB21 showed the highest activity at pH 7.0 and notable stability in acidic and alkaline conditions over a wide pH range (4–11) after 1 h incubation (Figure 2B).

### Adsorption rate and one-step growth assay

**Figure 3:**
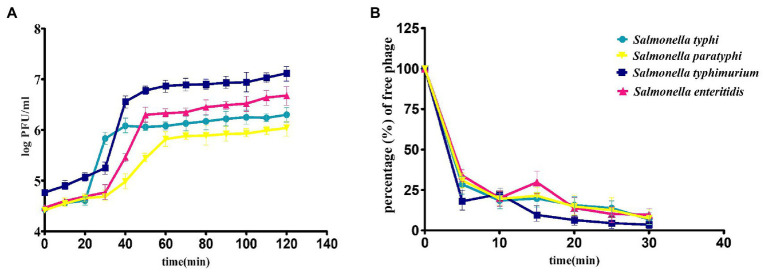
**(A)** One-step growth curve, **(B)** adsorption assay of bacteriophage STWB21 against *S. typhi, S. paratyphi, S. typhimurium* and *S. enteritidis*. Given values are the mean of three determinations.

Intracellular lytic development and the growth kinetics of phage STWB21 were analyzed in one-step growth assay on different typhoidal and non-typhoidal *Salmonella* species including host bacteria (Figure 3A). The latent period/burst size for *S. typhi, S. paratyphi, S. typhimurium* and *S. enteritidis* was found to be 25 min/101, 55 min/53, 50 min/163, 35 min/224 pfu, respectively, (Supplementary Table S2). Additionally, rapid adsorption was occurred for different typhoidal and non-typhoidal *Salmonella* species in first 5 min, followed by a slower adsorption phase thereafter (Figure 3B).

### Biofilm degradation assay and scanning electron microscopy analysis in coverslip, onion, and milk

**Figure 4:**
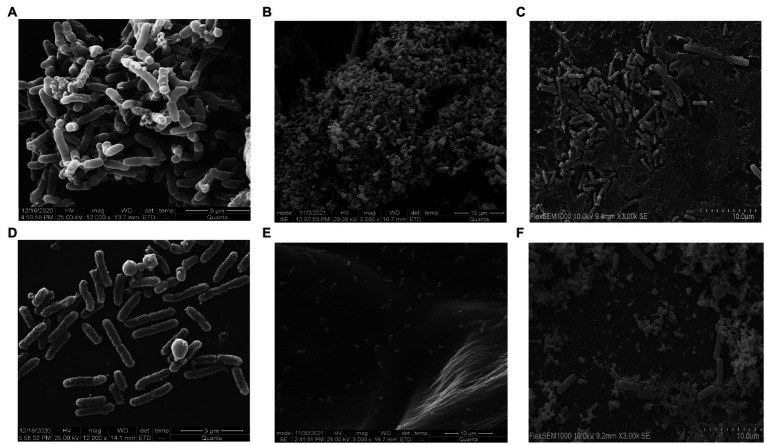
Scanning electron micrographs of *Salmonella typhi* biofilm formation and degradation assay. **(A, C, E)** show 24 h biofilm on coverslip, onion and in milk, respectively. **(B, D, F)** reveal the effect of bacteriophage STWB21 on biofilms formed on coverslip, onion and in milk, respectively.

*Salmonella typhi* culture was kept at 37°C in static condition to form bacterial biofilm of 24 h and treated with phage STWB21 to examine its ability to degrade biofilm. The experiment was done in coverslip, onion and milk. Scanning electron microscope analysis revealed a clear indication of *Salmonella typhi* biofilm formation on coverslip, onion and milk (Figures 4A,C,E) respectively. Once the biofilm was treated with phage STWB21 the bacterial population in biofilm was notably decreased on coverslip, onion and milk (Figures 4B,D,F) respectively.

### Quantification of *Salmonella* biofilm in microtiter plate

**Figure 5:**
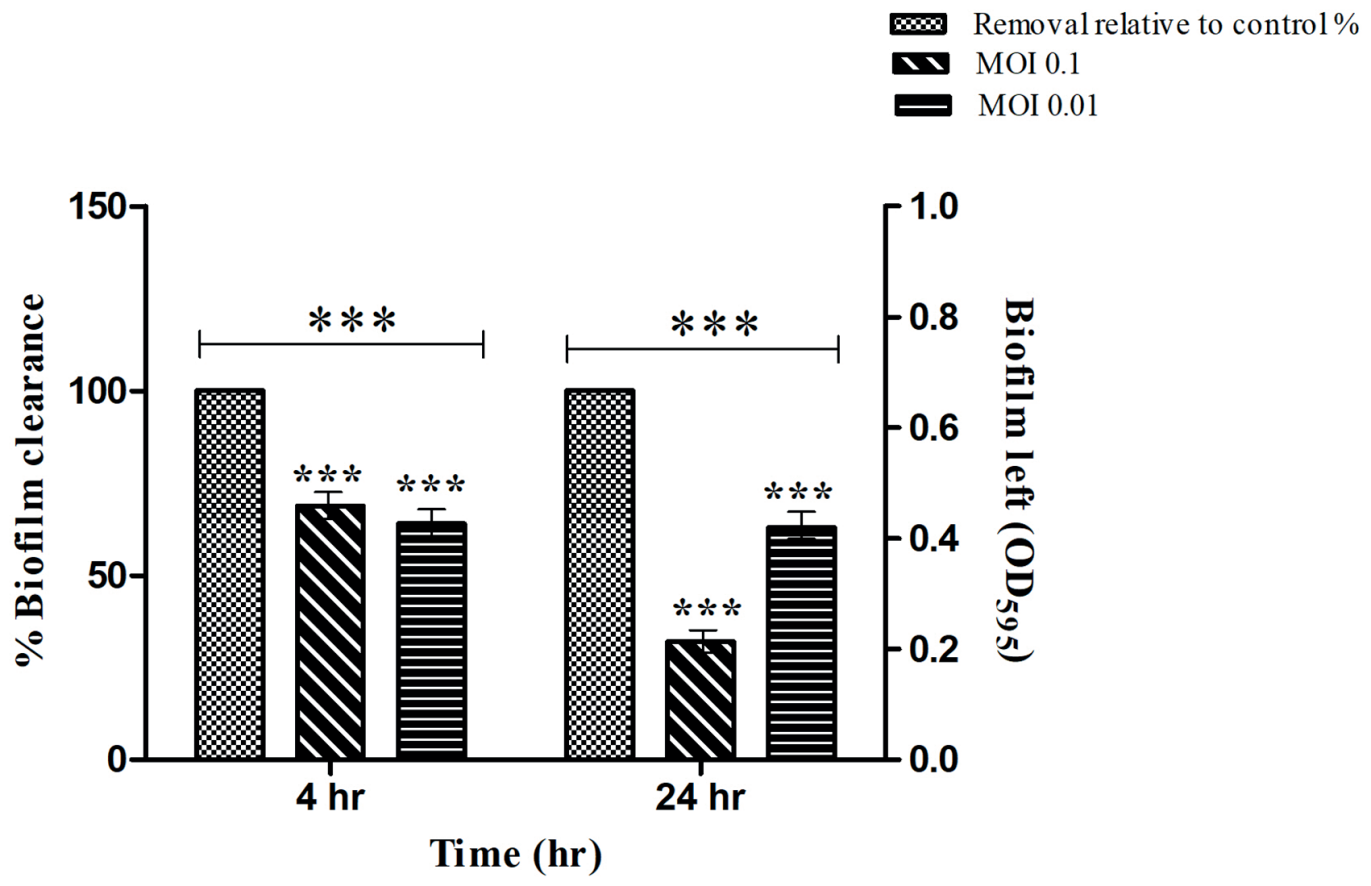
Quantification of *Salmonella* biofilm in Microtiter Plate. A 24 h biofilm of *S. typhi* formed in 96-well plates was treated with phage STWB21 at m.o.i 0.1 and 0.01 concentrations both at 4 h and 24 h time points. All the values were tabulated as mean ± SD and a significant difference between variations was denoted by asterisks (***p < 0.001) using two-way ANOVA.

The mature biofilm of *S. typhi* strain was significantly reduced by STWB21 phage. As shown in Figure 5, biofilm formation was significantly decreased (*p* < 0.001) by phage STWB21 at different concentrations (MOI 0.1 and 0.01). After 4 h, phage STWB21 removed 31% (*p* < 0.001) and 36% (*p* < 0.001) of the biofilm biomass at MOIs 0.1 and 0.01 respectively, when compared to control. However, after 24 h treatment the above-mentioned values changed to 68% (*p* < 0.001) and 37% (*p* < 0.001) respectively.

### Analysis of structural proteins of phage STWB21 by SDS-page

**Figure 6:**
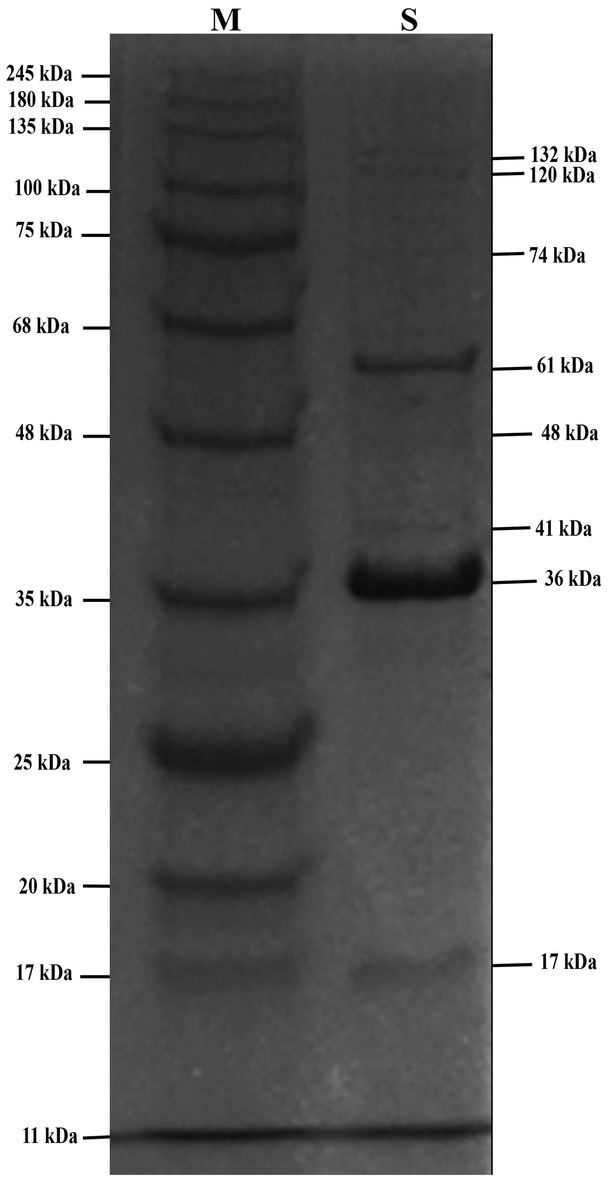
The SDS-PAGE analysis of phage STWB21 structural proteins on 12.5% gel staining with Coomassie brilliant blue. M, a standard marker of molecular weight (kDa).

SDS-PAGE analysis of phage STWB21 showed the presence of structural proteins ranging from 10 to 150 kDa. There are only two major bands that appeared at around 36 and 61 kDa while the visible minor bands appeared at 17, 41, 48, 74, 120 and 132 kDa (Figure 6).

### Proteomic features

To identify the structural proteins in phage STWB21, purified phage particles were analyzed by high-resolution Nano LC–MS/MS. A detailed proteomic characterization identified 19 proteins in phage STWB21 (Table 2). The structural proteins that were identified in phage STWB21 are six tail proteins, three tail fiber proteins, one prohead protease protein, one receptor-binding tail protein, one major capsid protein, one portal protein, and one capsid and scaffold protein. Interestingly, DNA metabolism module-related gene (A1 protein) and host lysis module-related gene (lysozyme) were identified. It is believed that the expression of A1 gene in T5 phage is required for the second step transfer of DNA.

**Table 2:** Identification of the phage STWB21 proteins by LC_MS/MS.

**ORF**	**Protein**	**Molecular mass (Da)**	**No. of peptides**	**Sequence coverage (%)**
1. ORF146	Tail protein	132,066	35	40
2. ORF151	L-shaped tail fiber	114,851	31	38
3. ORF142	Major tail protein	50,322	21	57
4. ORF148	Tail protein	107,179	20	30
5. ORF135	Tail fiber protein	16,967	16	82
6. ORF152	Tail fiber protein	75,110	8	20
7. ORF139	Hypothetical protein	19,405	8	71
8. ORF118	A1 protein	61,470	9	22
9. ORF149	Tail protein	74,758	5	9
10. ORF97	Capsid and scaffold protein	18,742	9	42
11. ORF137	Major capsid protein	23,016	6	11
12. ORF143	Hypothetical protein	34,437	6	34
13. ORF134	Portal protein	32,976	6	28
14. ORF150	Tail protein	15,508	5	49
15. ORF136	Prohead protease	23,372	6	38
16. ORF128	Receptor binding tail protein	64,149	2	6
17. ORF112	Hypothetical protein	36,314	4	21
18. ORF80	Lysozyme	15,236	3	27
19. ORF147	Tail protein	22,658	2	5

### Restriction profile of phage DNA

Phage STWB21 genome was cut with restriction enzymes EcoRI, EcoRV, MluI, BglII, PstI, BamHI, and HindIII (Supplementary Figure S1). Digestion of STWB21 genetic material with restriction enzymes confirmed that it is a double-stranded DNA virus.

### General features of the STWB21 genome

The complete genome length of phage STWB21 is 112,834 bp with a 40.37% GC content very similar to the *Salmonella* phages of T5 virus family: *Salmonella* phage S124, Seafire, Gec_vB_N3, S133, Seabear, Stitch (Table 3). Thus, according to Megablast results, STWB21 was enumerated as a member of the T5 virus genus, *Siphoviridae* subfamily.

**Table 3:** List of reported *Salmonella* phages from *Siphoviridae* family.

**Phage name**	**Similarity**	**Length**	**GC content**	**tRNA**	**Host**	**Taxonomic genera**	**Accession number**	**Reference**
STWB21	–	112,834	40.39%	22	*S. typhi*	T5 virus	MW567727	Present study
S124	92.69%	112,564	40.12%	28	*S. enterica* subsp.	T5 virus	NC_048013	NCBI Database (Unpublished)
Seafire	92.68%	111,851	40.01%	27	*S. enteritidis*	T5 virus	NC_048110	Hartman et al. 2019
Gec_vB_N3	92.40%	109,645	40.08%	17	*S. enteritidis*	T5 virus	MW006478	Makalatia et al. 2020
S133	92.28%	110,926	40.06%	29	*S. enterica* subsp.	T5 virus	NC_048011	NCBI Database (Unpublished)
Sea bear	92.08%	112,472	40.38%	25	*S. typhimurium*	T5 virus	MK728824	Patil et al. 2019
Stitch	91.90%	123,475	40.31%	30	*S. typhimurium*	T5 virus	KM236244	Grover et al. 2015

Phage STWB21 genome is predicted to encode 166 putative open reading frames (47 on the complementary strand and 119 on the direct strand) with 152 ATG (91.07%), 10 GTG (6.02%) and 5 TTG (2.99%) as initiation codons. After genome analysis, 22 tRNA encoding genes were found to be present in phage STWB21 and it seems to be a characteristic of a virulent phage. Closely related *Siphoviridae Salmonella* phages were also reported to carry a similarly large number of tRNA encoding genes (Table 3).

Phage STWB21 whole genome sequence was compared against the nucleotide sequence database in NCBI using BLASTn, Phage STWB21(MW567727) showed high homology with six bacteriophages: phage S124 (NC_048013), Seafire (NC_048110), Gec_vB_N3 (MW006478), S133 (NC_048011), Seabear (MK728824) and Stitch (KM236244). The detailed genomic features of the above-mentioned phages were listed in Table 3. The genomic sequence of STWB21 is 92.6% identical to that of S124, 92.6% to Seafire, 92.4% to Gec_vB_N3, 92.2% to S133, 92% to Seabear and 91.9% to Stitch as shown in Table 3.

**Figure 7:**
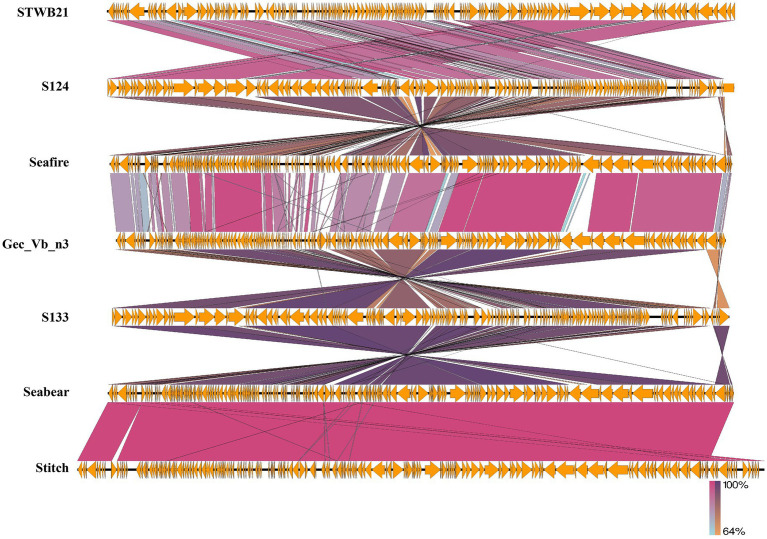
Genomic comparison of the phage STWB21 with other six homologous *Salmonella* phages using Easyfig application. Homologous ORFs or genes are present in yellow, and the percentages of amino acid identities are shown in different colors. Arrows indicate ORFs with either rightward or leftward direction.

By using the Easyfig genome comparison tool, we have compared phage STWB21 genomic synteny with other related *Siphoviridae Salmonella* phages: S124, Seafire, Gec_vB_N3, S133, Seabear, Stitch (Figure 7). Multiple alignment of phage STWB21 with other six relative phages have shown that the gene inventories of these six closely related phages are highly similar. As indicated in Figure 7, the genomes of the phages contain a block of clustered genes encoding predicted structural and functional proteins. In some cases, they were arranged differently and even oriented in opposite directions.

**Figure 8:**
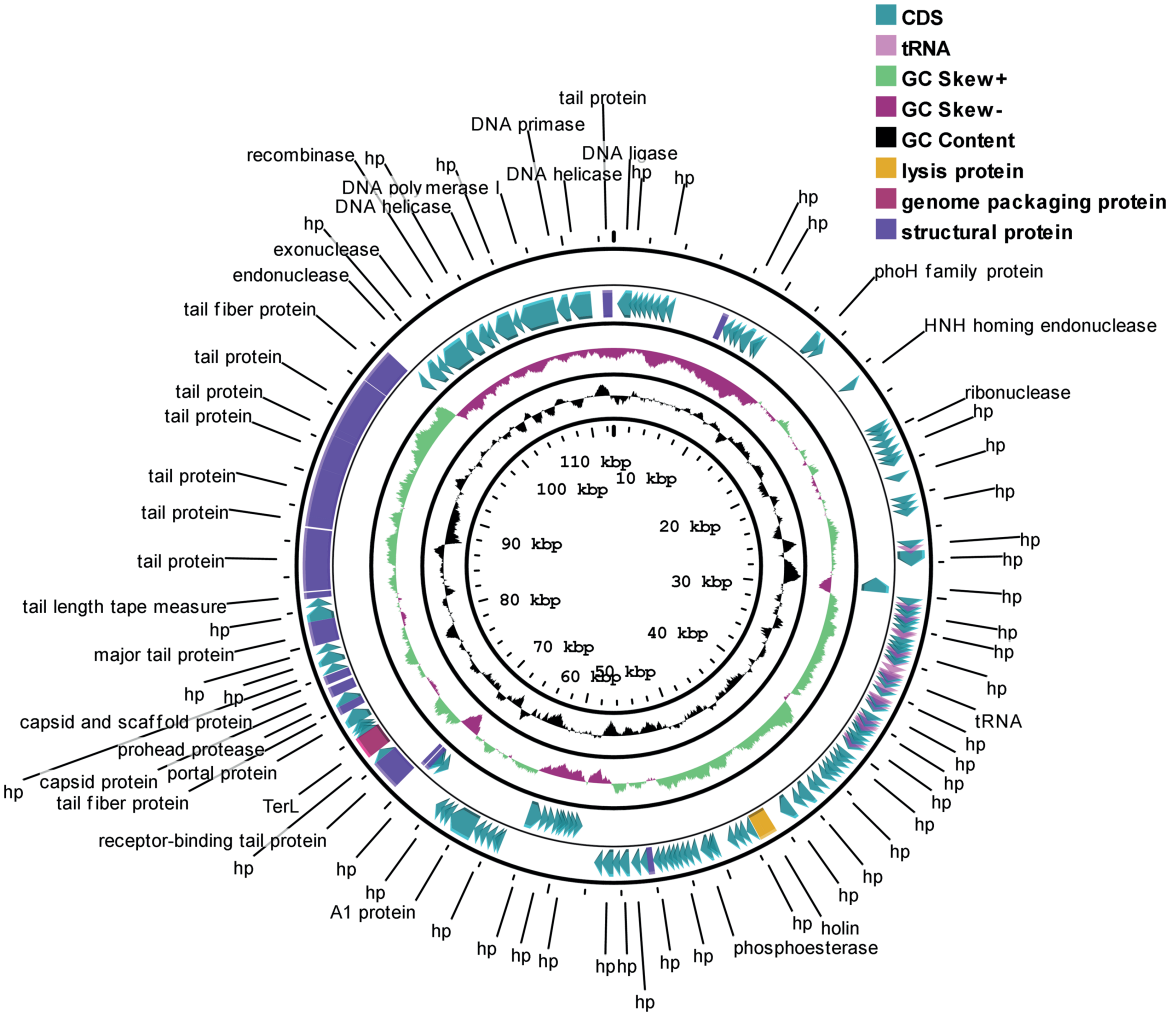
Genome structure of phage STWB21 prepared using CGView. The outer ring denotes the STWB21 genome and ORFs. The inner rings show G + C content and G + C skew, where peaks represent the positive (outward) and negative (inward) deviation from the mean G + C content and G + C skew, respectively.

As shown in Figure 8, the ORFs of STWB21 were broadly scattered across the genome and the gene annotation of predicted ORFs is listed in Supplementary Table S3.

### Description of phage modules

The annotated proteins of STWB21 can be categorized into four different genetic modules, which enable the following: DNA replication/modification/transcriptional regulations, lysis, DNA packaging and morphogenesis.

#### DNA replication/modification/transcriptional regulation module proteins

STWB21 encodes at least 12 genes co-localized as a distinct DNA modification module and were anticipated to play role in phage DNA metabolism, including a DNA helicase (ORF 160), a helicase-primase (ORF 163-ORF 164), a DNA polymerase I (ORF 162), a transcriptional factor (ORF 165), DNA ligase (ORF 1), transcriptional regulator/Methyltransferase (ORF 3), HNH homing endonuclease (ORF 21), ribonuclease (ORF 27), exonuclease (ORF 156), recombinase (ORF 157). The highly presence of DNA metabolism-associated genes in STWB21 genome might reduce the dependence of phage on bacteria (Peng and Yuan, 2018).

#### Two-component host lysis module proteins

A classic eubacterial phage lysis cassette compromises two-component cell lysis proteins, a holin (ORF 79) and lysozyme (ORF 80) are present in STWB21 genome. During the burst step of phage life cycle, these genes are crucial to effect host-cell lysis. Pore-forming enzyme holin permeabilizes the cytoplasmic membrane and cell wall degrading protein lysozyme degrades the bacterial cell wall (Wang et al., 2000). According to previous reports, holin have usually two or three transmembrane (Bläsi et al., 1999) domains but the genome analysis revealed that the holin of STWB21 has one transmembrane domain group (TMHMM-2.0). Besides, genomic analysis of phage STWB21 was studied for the presence of phage lysogeny factors and toxin genes. The presence of the lysis gene and the absence of lysogeny-related genes in the STWB21 genome clearly indicates that the phage is a potent lytic phage.

#### DNA packaging module proteins

In the DNA packaging modules, only ORF 130 was predicted which encodes the large subunit of terminase in the STWB21 genome and displayed 99% similarity with other *Salmonella* phage S124. Previous studies suggested that the large subunit of terminase is very conserved among the related phages (Burroughs et al., 2007). The junction of the DNA packaging module and the morphogenesis module is constituted by portal protein.

#### Phage morphogenesis module proteins

The arrangements of 22 genes encoding STWB21 phage structural proteins were scattered in the complementary strand of the genome and followed the gene orders of *Siphovirus*. These 22 ORFs mainly encode proteins like portal protein (ORF 133, ORF 134), tail fiber protein (ORF 135, ORF 151, ORF 152), major capsid protein (ORF 137), capsid and scaffold protein (ORF 138), tail length-tape measure protein (ORF 145), tail completion protein (ORF 166). As previously described, tail fiber proteins are subject to horizontal gene transfer between phages in a constant manner (Greive et al., 2016). During infection, tail length tape measure protein DNA is transited to the bacterial cell by the tape measure protein (Mahony et al., 2016).

### Phylogenetic analysis

**Figure 9:**
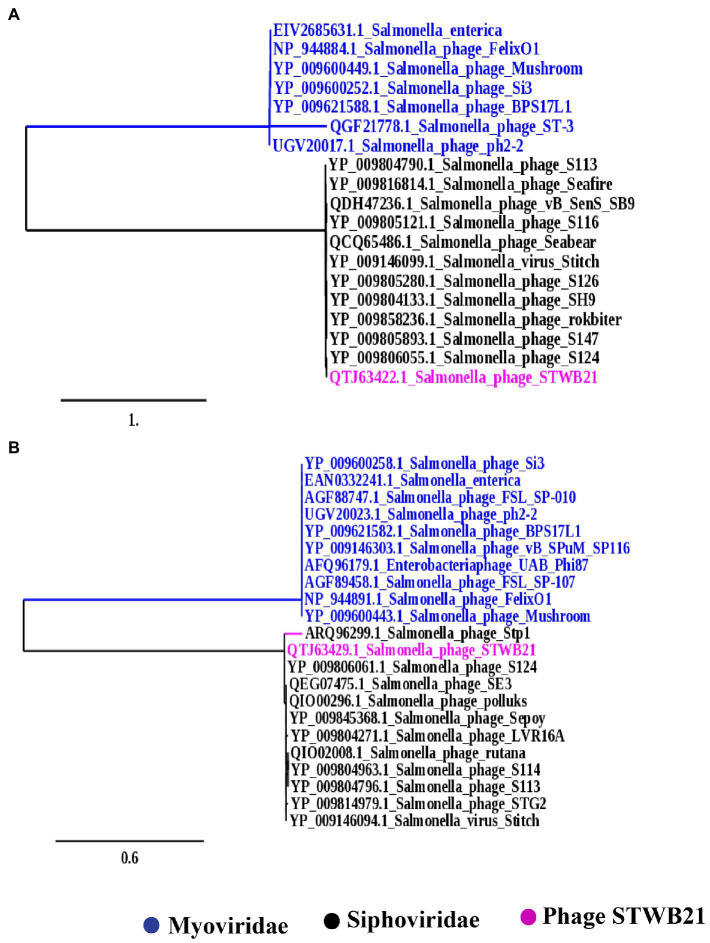
Phylogenetic analysis **(A)** large terminase unit, **(B)** major capsid proteins of *Salmonella* phage STWB21 with phages belonging to T4 and T5 family constructed using “One Click” at phylogeny.

To further understand the phylogenetic relationship of STWB21 to other *Salmonella* phages, two different types of phylogenetic trees were generated as shown in Figures 9A,B based on the terminase large subunit (TerL) and the major capsid protein, respectively. The tree showed that the phage STWB21 cluster together with T5-like *Salmonella* phages, such as S124, Stitch, SH9, S113, Sea bear, SE3 and Sepoy. Therefore, the investigated phage was considered as a new species of the T5 genus.

## Discussion

*Salmonella* genus is considered one of the biggest public threats due to its expanded reservoir (WHO, 2017). In addition, antibiotic resistance amidst bacterial infection has become a global burden (Tacconelli et al., 2018). To overcome this situation an alternative approach to treat bacterial infection is a pressing priority. Bacteriophages especially lytic bacteriophages can be effective against specific bacterial infections due to their ability to infect and kill specific host bacteria. Therefore, an upsurge in researches on bacteriophage isolation, characterization and effectiveness against pathogenic bacteria has reflected the awareness worldwide. Effective phage therapy requires specific conditions such as a large collection of bacteriophages, the use of obligately lytic phages rather than the temperate phage, host range, and screening of phage genomes to confirm the absence of toxin genes (Weber-Dabrowska et al., 2016). Furthermore, it is advantageous to use phages that adsorb efficiently on host cells, propagate rapidly with a relatively high burst size and may eliminate target bacteria in a relatively short time. Here, we reported the isolation and characterization of phage STWB21 and evaluated its application in food samples.

In this study, we have characterized a lytic bacteriophage STWB21 isolated from the lake water sample in the outskirts of Kolkata area using a strain of *S. typhi* as the host bacterium. Phage STWB21 was found to be effective against many other strains of typhoidal and non-typhoidal *Salmonella* which indicates the high specificity of STWB21 against *Salmonella* (Abdelsattar et al., 2021). On the other hand, the ability of phage STWB21 to lyse *Sh. flexneri 2a*, *Sh. flexneri 3*, and *ETEC* indicate that the phage can infect more than one bacterial species. Thus, phage STWB21 is a “polyvalent phage,” as this quality has been reported before for other *Salmonella* phages (Parra and Robeson, 2016). In addition, to the best of our knowledge, this is the first *Salmonella* phage active against typhoidal strains as well as non-typhoidal strains.

Resistance to heat and acid–base properties are the foundations of phage used in bio-control applications (Jończyk-Matysiak et al., 2019). Different foods like fruits, vegetables, pasteurized milk or chicken breast are usually stored at 4°C or at ambient temperature (25°C). According to our results, the phage STWB21 was stable at 4°C to <50°C, and the stability of phage STWB21 at ambient temperature without losing viability for a month, implied its suitability for large-scale applications. The phage STWB21 is relatively stable within the range of pH between 4 and 11, which is congenial with the range (pH 5.5–7.0) of many foods. Therefore, their ability to survive at different temperature and pH conditions suggest that phage STWB21 is tolerant to heat and extreme pH conditions and it could be used in different food industries.

During October 2021, food poisoning outbreak news came out from California, New York, North Carolina, Pennsylvania and Texas among adults due to the consumption of imported whole red, white and yellow onions. There are published reports controlling the biofilm on coverslips, fruits, vegetables and pasteurized milk using bacteriophages (Isoken, 2015; Amrutha et al., 2017; Wang et al., 2018). However, no reports are available yet using phages that can control *Salmonella* spp. biofilm formation on the red onion. In this study, we evaluated the ability of lytic phage STWB21 to remove biofilm from glass coverslips, red onion and pasteurized milk. After treatment with phage STWB21, the number of bacteria in the biofilm was decreased significantly. Moreover, biofilms were considerably degraded, and disrupted and did not show any microbial regrowth. Therefore, anti-biofilm results support that phage STWB21 can be used as a “biological disinfectant” capable of controlling *Salmonella* infections.

In our study, detailed genomic and proteomic analyses of phage STWB21 revealed valuable information concerning its biology and showed a modular organization that is very similar to other T5-like *Salmonella* bacteriophages S124, Gec_vB_N3, S133, Seabear, Stitch (Grover et al., 2015; Patil et al., 2019; Necel et al., 2020). Following LC–MS/MS analysis, 17 structural proteins and 2 functional proteins have been identified. Moreover, genomic and proteomic analyses indicated that the phage STWB21 genome does not encode any toxin gene, antibiotic resistance gene, phage lysogeny factors or pathogen-related genes, suggesting that the phage STWB21 may be considered as a reliable phage therapy candidate with no side effects. Bacteriophage STWB21 forms clear small plaques without any halo as shown in Figure 1A. In addition, the presence of host lysis proteins (holin and lysozyme) in its genome confirms that phage STWB21 is a virulent lytic phage. *Salmonella* phages vB_Sen-TO17 and vB_Sen-E22 were also confirmed to be lytic in nature based on similar observations (Kwaśnicka et al., 2020).

The phage STWB21 harbors 22 tRNA genes corresponding to 14 different amino acids and has a significantly different codon usage than its host. Moreover, tRNA genes are usually considered as indispensable information-related and housekeeping genes which are least susceptible to lateral gene transfer (Grover et al., 2015). In a previous study on *Siphoviridae* phages, it was found that there is no clear correlation between the number of tRNAs and burst size or latent period (Ding et al., 2020). Our result also supports the previous study as phage STWB21 has 22 tRNA but showed different latent periods and burst sizes for typhoidal and nontyphoidal *Salmonella* spp. The phylogenetic analysis led to phage STWB21 clustering with T5-like phages rather than with T4-like phages.

## Conclusion and future work

This study elucidated the environmental isolation of polyvalent *Salmonella* bacteriophage STWB21 shown to be efficacious against a wide host range spectrum of human pathogenic enteric bacteria. STWB21 exhibited robust pH and thermal stability as well as biofilm degradation activity. Thus, these observations place phage STWB21 among candidate phages as an effective biocontrol agent. Our future studies on in-vivo phage application to control *Salmonella* infection will be conducted to understand more about the antimicrobial property of phage STWB21.

## Data availability statement

The complete genome sequence of phage STWB21 has been deposited in GenBank under accession no. MW567727.

## Author contributions

PM and MD conceived and designed the experiments. PM carried out the main body of research, performed the experiments and bioinformatics analysis, and wrote the manuscript. BM contributed in performing the phage characterization experiment. SD provided the typhoidal *Salmonella* strains and reviewed the manuscript. MD supervised the work and edited the manuscript. All authors contributed to the article and approved the submitted version.

## Funding

The work was supported by Institutional fund which has already been mentioned. So, the grant number is not applicable here.

## Conflict of interest

The authors declare that the research was conducted in the absence of any commercial or financial relationships that could be construed as a potential conflict of interest.

## Publisher’s note

All claims expressed in this article are solely those of the authors and do not necessarily represent those of their affiliated organizations, or those of the publisher, the editors and the reviewers. Any product that may be evaluated in this article, or claim that may be made by its manufacturer, is not guaranteed or endorsed by the publisher.

## Footnotes

^1^ http://exon.gatech.edu/GeneMark/index.html

^2^ http://lowelab.ucsc.edu/cgi-bin/tRNAscan-SE2.cgi

^3^ http://mjsull.github.io/Easyfig/files.html

^4^ https://www.ncbi.nlm.nih.gov
