# Supplementary material for: Isolation, characterization, and application of a novel polyvalent lytic phage STWB21 against typhoidal and nontyphoidal Salmonella spp
Source: Front Microbiol. 2022 Aug 22;13:980025. doi: 10.3389/fmicb.2022.980025 (PMC9441917; doi:10.3389/fmicb.2022.980025)
Supplement: Supplementary file 1 [file Data_Sheet_1.PDF]

Supplementary Material

Supplementary Figures

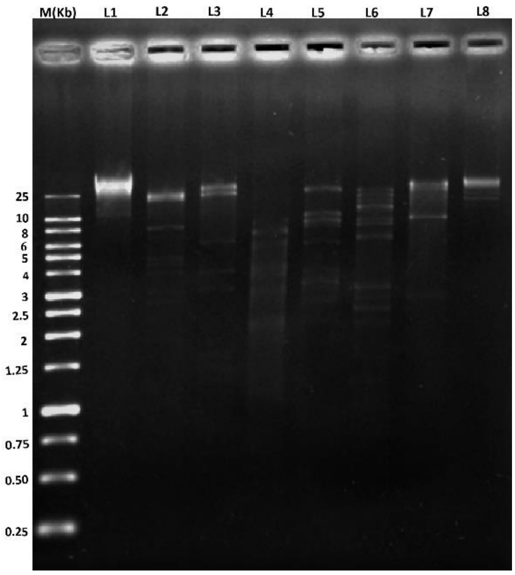

Supplementary Figure S1. Restriction fragment analysis of the phage STWB21 genomic DNA. Digestion pattern with EcoRI (L2), EcoRV (L3), MluI (L4), BglII (L5), PstI (L6), BamHI (L7), and HindIII (L8). Control: Genomic DNA (L1). M, Marker.

Supplementary Tables

Strains	Strain name	E. O. P
1. *S. typhi*	*(KOL 551) **	1
2. *S. typhi*	*(KOL 553)*	0.07
3. *S. typhi*	*(C6953)*	0.33
4. *S. typhi*	*(K432)*	0.06
5. *S. paratyphi*	*(KOL 534) **	0.51
6. *S. paratyphi*	*(KOL 539)*	0.30
7. *S. paratyphi*	*(KOL 520)*	0.34
8. *S. enteritidis*	*(520833) **	0.73
9. *S. typhimurium*	*(PH-94) **	0.81
10. *ETEC*	*(IDH07942)*	0.05
11. *ETEC*	*(IDH H10407)*	0.0002
12. *ETEC*	*(IDH H10404)*	-
13. *Sh. flexneri 2a*	*(2457T)*	0.016
14. *Sh. flexneri 3a*	*(UB811)*	0.004
15. *Sh. flexneri 6*	*(UB812)*	-
16. *Sh. boydii*	*(NK02379)*	-
17. *V. cholerae O1*	*(MAK757)*	-

Table S1. Efficacy of plating of phage STWB21 against different bacterial strains.
* Strains were used in this study.

Strain name	Latent phase	Burst size
Salmonella typhi	25 min	101 pfu/cell
Salmonella paratyphi	55 min	53 pfu/cell
Salmonella enteritidis	50 min	163 pfu/cell
Salmonella typhimurium	35 min	224 pfu/cell

Table S2. The latent phase and burst size of phage STWB21 on different typhoidal and non-typhoidal *Salmonella.*

	CDS position	Function	Blast hit
ORF 1	1195..221	DNA ligase	PHAGE_Salmon_S124_NC_048013
ORF 2	1388..1188	HP	PHAGE_Salmon_S131_NC_048009
ORF 3	1782..1474	Transcriptional factor (Methyl transferase)	PHAGE_Salmon_S124_NC_048013
ORF 4	2129..1833	HP	PHAGE_Salmon_S124_NC_048013
ORF 5	2577..2167	putative D3 protein	PHAGE_Salmon_bobsandoy_MT074464
ORF 6	2939..2685	DNA helicase	PHAGE_Salmon_S147_NC_048012
ORF 7	3636..2932	D2 protein	PHAGE_Salmon_bobsandoy_MT074464
ORF 8	3938..3705	HP	PHAGE_Salmon_vBSenS3_154_ MT004791
ORF 9	6711..3922	replication origin binding protein	PHAGE_Salmon_STG2_NC_048089
ORF 10	7742..7332	HNH endonuclease	PHAGE_Salmon_STG2_NC_048089
ORF 11	8182..7753	HP	PHAGE_Escher_fp01_NC_048731
ORF 12	8690..8184	exonuclease	PHAGE_Salmon_S132_NC_048010
ORF 13	8862..8677	capsid maturation protease	PHAGE_Salmon_faergetype_NC_048867
ORF 14	9779..8979	recombination protein	PHAGE_Salmon_S132_NC_048010
ORF 15	10189..9986	HP	PHAGE_Salmon_rokbiter_NC_048868
ORF 16	10480..10199	HP	PHAGE_Salmon_ende_75_MT074454
ORF 17	12453..10579	anaerobic ribonucleoside-triphosphate reductase	PHAGE_Salmon_Sw2_NC_048062
ORF 18	12806..13558	PhoH family protein	PHAGE_Salmon_enterica_ABBNMR010000014
ORF 19	13560..13802	HP	PHAGE_Salmon_S126_NC_048008
ORF 20	13866..16259	Ribonucleoside diphosphate	PHAGE_Salmon_vB_SenS-3_MT004791

		reductase I alpha chain	
ORF 21	16341..16838	HNH homing endonuclease	PHAGE_Salmon_vB_SenS-SB13_ NC_048781
ORF 22	16835..17980	Ribonucleotide reductase of class Ia beta subunit	PHAGE_Salmon_S124_NC_048013
ORF 23	17980..18516	Dihydrofolate reductase	PHAGE_Salmon_S124_NC_048013
ORF 24	18613..19367	Thymidylate synthase	PHAGE_Salmon_rokbiter_NC_048868
ORF 25	19460..19714	HP	PHAGE_Salmon_S133_NC_048005
ORF 26	19714..19983	HP	PHAGE_Salmon_sent65_MT653145
ORF 27	19983..20459	Ribonuclease	PHAGE_Salmon_S132_NC_048010
ORF 28	20536..20814	HP	PHAGE_Salmon_S124_NC_048013
ORF 29	20899..21414	HP	PHAGE_Salmon_S124_NC_048013
ORF 30	21476..21691	HP	PHAGE_Salmon_faergetype_NC_048867
ORF 31	21733..21939	HP	PHAGE_Salmon_S124_NC_048013
ORF 32	21965..22666	Metallopeptidase	PHAGE_Salmon_S124_NC_048013
ORF 33	22737..22919	Hp	PHAGE_Salmon_S126_NC_048008
ORF 34	22974..23612	Tail fiber protein	PHAGE_Salmon_Stitch_NC_027297
ORF 35	24054..24371	Hp	PHAGE_Salmon_vB_SenS_SB13_NC_048781
ORF 36	24377..24826	Cell wall hydrolyse	PHAGE_Salmon_S124_NC_048013
ORF 37	24895..25065	HP	PHAGE_Salmon_SE11_NC_048786
ORF 38	25065..25508	YqeY protein domain-containing protein	PHAGE_Salmon_falkor_MT074467
ORF 39	26674..27183	HP	PHAGE_Salmon_S124_NC_048013
ORF 40	27300..28244	HP	PHAGE_Salmon_OSY_STA_NC_048808
ORF 41	28980..28564	cAMP-dependent protein kinase catalytic subunit	PHAGE_Salmon_Sepoy_NC_048760

ORF 42	29995..29003	HP	PHAGE_Salmon_Sepoy_NC_048760
ORF 43	30261..30557	HP	PHAGE_Salmon_S126_NC_048008
ORF 44	30803..30988	HP	PHAGE_Salmon_S132_NC_048010
ORF 45	30990..31322	HP	PHAGE_Salmon_STG2_NC_048089
ORF 46	31508..31630	HP	PHAGE_Salmon_Stitch_NC_027297
ORF 47	31644..31850	HP HOT59_gp160	PHAGE_Salmon_S113_ NC_048005
ORF 48	31941..32216	HP	PHAGE_Salmon_Stitch_ NC_027297
ORF 49	32703..32975	HP HOT61_gp148	PHAGE_Salmon_S116_NC_048007
ORF 50	33026..33250	HP	PHAGE_Salmon_S113_NC_048005
ORF 51	33247..33519	HP	PHAGE_Salmon_S124_NC_048013
ORF 52	33714..33899	HP	PHAGE Salmon_Stitch_ NC_027297
ORF 53	34003..34161	HP HOS12_gp103	PHAGE_Salmon_SP01_NC_047859
ORF 54	35148..35312	HP	PHAGE_Salmon_falkor_MT074467
ORF 55	35411..35728	HP	PHAGE_Salmon_Stitch_NC_027297
ORF 56	35961..36314	HP BOW73_gp180	PHAGE_Salmon_100268_sal2_NC_031902
ORF 57	36773..36991	HP	PHAGE_Salmon_S116_NC_048007
ORF 58	37097..37270	HP	PHAGE_Salmon_Smaug_MT074461
ORF 59	37720..37899	HP	PHAGE_Salmon_Stitch_ NC_027297
ORF 60	38115..38315	HP	PHAGE_Salmon_ vB_SenS_SB9_ MK867835
ORF 61	38578..38799	HP	PHAGE_Salmon_Stitch_ NC_027297
ORF 62	38792..38956	HP	PHAGE_Salmon_Stitch_ NC_027297
ORF 63	39116..39409	HP	PHAGE_Salmon_faergetype_ NC_048867
ORF 64	39409..39609	HP	PHAGE_Salmon_Stitch_ NC_027297
ORF 65	39801..39974	HP	PHAGE_Salmon_vB_SenS-3_MT004791

ORF 66	39952..40320	Pyruvate formate lyase	PHAGE_Salmon_S113_NC_048005
ORF 67	40402..40689	HP	PHAGE_Salmon_S113_NC_048005
ORF 68	40754..40939	HP	PHAGE_Salmon_faergetype_NC_048867
ORF 69	40998..41393	HP	PHAGE_Salmon_faergetype_NC_048867
ORF 70	41470..41742	HP	PHAGE_Salmon_rokbiter_NC_048868
ORF 71	41742..42041	HP	PHAGE_Salmon_atrejo_NC_048872
ORF 72	42034..42453	HP	PHAGE_Salmon_vb Sens-3_MT004791
ORF 73	42697..42981	HP	PHAGE_Salmon_STG2_NC_048089
ORF 74	43036..43449	HP	PHAGE_Salmon_S124_NC_048013
ORF 75	43598..44296	HP	PHAGE_Salmon_rokbiter_NC_048868
ORF 76	44253..44702	HP	PHAGE_Salmon_faergetype_NC_048867
ORF 77	44986..45738	HP	PHAGE_Salmon_OSY_STA_NC_048808
ORF 78	45751..46350	ATP-dependent Clp protease	PHAGE_Salmon_rokbiter_NC_048868
ORF 79	46507..47163	Holin	PHAGE Salmon_Stitch_ NC_027297
ORF 80	47160..47573	Lysozyme/endolysin	PHAGE Salmon_Stitch_ NC_027297
ORF 81	47649..48065	HP	PHAGE_Salmon_faergetype_NC_048867
ORF 82	48139..48549	HP	PHAGE_Salmon_faergetype_NC_048867
ORF 83	48542..48832	Thioredoxin	PHAGE_Salmon_S124_NC_048013
ORF 84	48935..49315	HP	PHAGE_Salmon_S124_NC_048013
ORF 85	49315..50178	Serine threonine phosphatase	PHAGE_Salmon_S124_NC_048013
ORF 86	50178..50384	HP	PHAGE_Salmon_S113_ NC_048005
ORF 87	50384..50482	HP	PHAGE Salmon_ vB_SenS_SB6_ MK809530
ORF 88	50492..51115	Phosphoesterase	PHAGE Salmon_ vB_SenS_ SB6_ MK809530
ORF 89	51277..51708	HP	PHAGE_Salmon_S124_NC_048013

ORF 90	51787..52038	HP	PHAGE_Salmon_S126_NC_048008
ORF 91	52038..52445	HP	PHAGE_Salmon_Sw2_NC_048062
ORF 92	52442..52723	HP	PHAGE_Salmon_S124_NC_048013
ORF 93	52720..52965	HP	PHAGE_Salmon_1_23_NC_048149
ORF 94	52955..53284	HP	PHAGE_Salmon_1_23_NC_048149
ORF 95	53385..53582	HP	PHAGE_Salmon_1_23_NC_048149
ORF 96	53579..54046	Hp	PHAGE_Salmon_OSY_STA_NC_048808
ORF 97	54021..54371	Capsid and scaffold protein	PHAGE_Salmon_bobsandoy_MT074464
ORF 98	54382..54804	HP HOT59 gp107_	PHAGE_Salmon_S113_ NC_048005
ORF 99	54826..55374	HP	PHAGE_Salmon_S132_NC_048010
ORF 100	55480..56136	HP	PHAGE_Salmon_vB_SenS_SB13_NC_048781
ORF 101	56136..56444	HP	PHAGE_Salmon_Seafire_NC_048110
ORF 102	56441..57169	HP	PHAGE_Salmon_1_23_NC_048149
ORF 103	57295..57435	HP	PHAGE_Salmon_Seafire_NC_048110
ORF 104	58946..58707	HP	PHAGE_Salmon_2_3_NC_048809
ORF 105	59286..59074	Methyl transferase	PHAGE_Salmon_OSY_STA_NC_048808
ORF 106	59627..59283	Hp	PHAGE_Salmon_S124_NC_048013
ORF 107	59873..59751	Hp	PHAGE_Salmon_bobsandoy_MT074464
ORF 108	60082..59870	HP	PHAGE Salmon_Stitch_ NC_027297
ORF 109	60363..60136	HP	PHAGE_Salmon_SH9_NC_047999
ORF 110	61016..60471	HP	PHAGE_Salmon_STG2_NC_048089
ORF 111	61339..61031	HP	PHAGE_Salmon_SH9_NC_047999
ORF 112	62395..61397	HP	PHAGE_Salmon_S114_NC_048006
ORF 113	63487..63645	HP	PHAGE_Salmon_Seafire_NC_048110

ORF 114	63645..63851	HP	PHAGE_Salmon_S114_NC_048006
ORF 115	64023..64274	HP	PHAGE_Salmon_S126_NC_048008
ORF 116	64374..64790	A2 protein	PHAGE_Salmon_S113_NC_048005
ORF 117	64841..65065	HP	PHAGE_Salmon_vB_SenS_SB13_NC_048781
ORF 118	65175..66842	A1 protein	PHAGE_Salmon_S124_NC_048006
ORF 119	66896..67156	HP	PHAGE_Salmon_SH9_NC_047999
ORF 120	67169..67447	HP	PHAGE_Salmon_bux_MT074460
ORF 121	67481..67873	HP	PHAGE_Salmon_fuchur_NC_048869
ORF 122	67957..68691	Deoxynucleoside-5-monophosphatase	PHAGE Salmon_Stitch_ NC_027297
ORF 123	69064..68849	HP	PHAGE Salmon_Stitch_ NC_027297
ORF 124	69493..69299	HP	PHAGE_Salmon_fuchur_NC_048869
ORF 125	69597..69493	HP	PHAGE_Salmon_S116_NC_048007
ORF 126	69984..69670	Receptor-blocking tail protein	PHAGE_Salmon_S124_NC_048006
ORF 127	70325..70059	Receptor-blocking tail protein	PHAGE Salmon_Stitch_ NC_027297
ORF 128	70409..72190	Receptor-binding tail protein	PHAGE_Salmon_fuchur_NC_048869
ORF 129	72201..72683	HP	PHAGE_Salmon_S124_NC_048013
ORF 130	72683..73999	Terminase large subunit	PHAGE_Salmon_S124_NC_048013
ORF 131	74114..74287	HP	PHAGE_Salmon_SP3_NC_048001
ORF 132	74373..74513	Hp	PHAGE_Salmon_S124_NC_048013
ORF 133	74513..74731	Portal protein	PHAGE_Salmon_S131_NC_048009
ORF 134	74739..75623	Portal protein	PHAGE_Salmon_S124_NC_048013
ORF 135	75620..76102	Tail fiber protein	PHAGE_Salmon_Sw2_NC_048062
ORF 136	76106..76738	Prohead protease	PHAGE_Salmon_S147_NC_048012
ORF 137	76756..77370	Major capsid protein	PHAGE_Salmon_S124_NC_048013

ORF 138	77549..78064	Capsid and scaffold protein	PHAGE_Salmon_S124_NC_048013
ORF 139	78124..78636	HP	PHAGE_Salmon_S124_NC_048013
ORF 140	78636..79403	HP	PHAGE_Salmon_S124_NC_048013
ORF 141	79407..79892	HP	PHAGE_Salmon_S124_NC_048013
ORF 142	79919..81328	Major tail protein	PHAGE_Salmon_S124_NC_048013
ORF 143	81333..82229	HP	PHAGE_Salmon_S124_NC_048013
ORF 144	82226..82630	HP	PHAGE_Salmon_S124_NC_048013
ORF 145	82692..83060	Tail length tape-measure protein	PHAGE_Salmon_S124_NC_048013
ORF 146	83141..86821	Tail protein	PHAGE_Salmon_S124_NC_048013
ORF 147	86930..87540	Tail protein	PHAGE_Salmon_S124_NC_048013
ORF 148	87541..90390	Tail protein	PHAGE_Salmon_SH9_NC_047999
ORF 149	90391..92448	Tail protein	PHAGE_Salmon_S124_NC_048013
ORF 150	92454..92876	Tail protein	PHAGE_Salmon_S124_NC_048013
ORF 151	92876..96112	L-shaped tail fiber	PHAGE_Salmon_Stitch_NC_027297
ORF 152	96153..98243	Tail fiber protein	PHAGE_Salmon_vb Sens-3_MT004791
ORF 153	98534..98280	HP	PHAGE_Salmon_S131_NC_048009
ORF 154	99830..98995	Endonuclease	PHAGE_Salmon_Sw2_NC_048062
ORF 155	100312..99830	D 14 protein	PHAGE_Salmon_Sw2_NC_048062
ORF 156	102153..100315	Exonuclease	PHAGE_Salmon_Polluks_MT074456
ORF 157	103111..102134	Recombinase	PHAGE_Salmon_SH9_NC_047999
ORF 158	103924..103151	HP	PHAGE_Salmon_SH9_NC_047999
ORF 159	104201..103917	HP	PHAGE_Salmon_S114_NC_048006
ORF 160	105774..104422	DNA helicase	PHAGE Salmon_ vB_SenS_SB9_ MK867835
ORF 161	106268..105771	HP	PHAGE_Salmon_fuchur_NC_048869

ORF 162	108828..106261	DNA polymerase I	PHAGE_Salmon_atrejo_NC_048872
ORF 163	109781..108891	DNA primase	PHAGE_Salmon_S124_NC_048013
ORF 164	111301..109778	DNA helicase	PHAGE_Salmon_S124_NC_048013
ORF 165	112100..111333	Transcriptional factor	PHAGE_Salmon_S124_NC_048013
ORF 166	112737..112093	Tail completion protein	PHAGE_Salmon_S124_NC_048013

Table S3. Features of the open reading frames (ORFs) of bacteriophage STWB21 and homology to protein database.
